# The Diabetes–Viral Respiratory Syndemic: Pathophysiological Insights and Precision Management: A Scoping Review

**DOI:** 10.3390/medicina62040770

**Published:** 2026-04-16

**Authors:** Ana Maria Mihai, Monica Marc, Florina Lucaciu, Alexandra Sima

**Affiliations:** 1 Doctoral School, Faculty of Medicine, Victor Babes University of Medicine and Pharmacy, 300041 Timisoara, Romania; ana-maria.mihai@umft.ro (A.M.M.); florina.lucaciu@umft.ro (F.L.); 2 Department of Infectious Diseases, Clinical Hospital of Infectious Diseases and Pulmonology “Victor Babes”, Gheorghe Adam Street 13, 300310 Timisoara, Romania; 3 Center for Research and Innovation in Personalized Medicine of Respiratory Diseases, Victor Babes University of Medicine and Pharmacy Timisoara, Eftimie Murgu Square 2, 300041 Timisoara, Romania; 4 Department of Diabetes, “Pius Brinzeu” Emergency Hospital, 300723 Timisoara, Romania; sima.alexandra@umft.ro; 5 Second Department of Internal Medicine, Faculty of Medicine, Victor Babes University of Medicine and Pharmacy, 300041 Timisoara, Romania

**Keywords:** diabetes mellitus, euglycemic diabetic ketoacidosis, immunothrombosis, innate immune dysfunction, metabolic memory, syndemic, stress hyperglycemia ratio, sodium–glucose transporter 2 inhibitors, viral respiratory tract infections

## Abstract

*Background/Objectives:* Viral respiratory tract infections (VRTIs) in patients with diabetes mellitus (DM) are characterized by a severity gap rather than an infection gap. This review synthesizes evidence from the 2023–2026 respiratory seasons to provide a post-pandemic framework for managing the synergistic metabolic and viral threats in this population. *Materials and Methods:* A scoping review of literature from PubMed, Scopus, and Embase (2023–2026) was conducted, focusing on clinical outcomes and mechanistic interactions between DM and emerging respiratory pathogens. *Results:* Recent data identify human Metapneumovirus (hMPV) and adenovirus as significant threats to diabetic hosts, with mortality risks equivalent to seasonal influenza (HR 1.00 for hMPV vs. influenza). The two-hit model combines a baseline of innate immune paralysis, characterized by impaired neutrophil chemo-taxis and mechanical SP-D dysfunction, with a cellular signaling environment primed for cytokine overreaction by epigenetic metabolic memory. The stress hyperglycemia ratio (SHR) has emerged as a promising predictor of mortality compared to absolute glucose or HbA1c, with a proposed threshold of ≥1.14 identifying patients at 3.5-fold increased risk for mechanical ventilation. Precision management should consider the prudent suspension of SGLT2 inhibitors to mitigate euglycemic DKA risks and considering the early use of GLP-1 receptor agonists for their hypothesized pulmonary anti-inflammatory properties. *Conclusions:* Closing the mortality gap may require a shift from generic viral care to a precision model that treats metabolic susceptibility with high clinical priority alongside the treatment of the viral pathogen.

## 1. Introduction

Viral respiratory tract infections (VRTIs), including seasonal influenza, RSV, and emerging agents like SARS-CoV-2, represent a continuous global threat [1,2]. Diabetes stands as a primary driver of non-transmissible disease burden, with cases predicted to rise to 72.4 million in Europe by 2050 [3]. While infection rates are often similar between diabetic and non-diabetic populations, the diabetic cohort is significantly more likely to develop severe symptoms and critical illness. Every year, influenza alone causes epidemics affecting millions of people worldwide, leading to a surge in medical visits and hospitalization during the peak season [1]. Unfortunately, vaccination rates in most countries remain well below the 75% recommended by the World Health Organization. Diabetic populations have a higher prevalence of vaccine hesitancy compared with non-diabetic populations, numerous studies report reduced influenza vaccination coverage and elevated hesitancy in this population [4].

In this review, the “post-pandemic era” is defined as the period beginning in May 2023, following the World Health Organization’s (WHO) official declaration terminating the Public Health Emergency of International Concern for COVID-19. This period (2023–2026) is characterized by the resurgence of non-COVID respiratory pathogens and a shifted immunological landscape in metabolically compromised hosts.

Viral respiratory tract infections (VRTIs) remain a persistent threat to global health, but for the population with diabetes mellitus (DM), the risk is uniquely lethal. Infection rates between diabetic and non-diabetic cohorts are often comparable; the conversion rate from mild illness to critical respiratory failure is significantly higher in the diabetic population. This severity gap represents a syndemic where metabolic toxicity and viral virulence amplify one another.

In the post-pandemic era (2023–2026), the clinical landscape has shifted [5]. Pathogens previously considered pediatric or opportunistic, such as hMPV and adenovirus, have emerged as primary drivers of severe lower respiratory tract infections in metabolically compromised adults. DM has been isolated as an independent risk factor for progression to Acute Respiratory Distress Syndrome (ARDS) [6] and Acute Kidney Injury (AKI); in these infections, with mortality risks mirroring or exceeding those of seasonal influenza [7].

The underlying susceptibility stems from a two-hit pathophysiological model: a baseline of innate immune paralysis (impaired neutrophil chemotaxis and SP-D dysfunction) coupled with a second hit of acute viral stress that triggers euglycemic ketoacidosis and metabolic memory flares. The introduction of newer glucose-lowering agents, such as SGLT2 inhibitors, has introduced novel iatrogenic risks like euglycemic DKA (euDKA) during acute viral stress.

Despite these insights, clinical management often remains fragmented. This review aims to integrate recent (2023–2026) scientific evidence to propose an updated framework for the precision management of VRTIs in DM. We focus on the superior prognostic utility of the stress hyperglycemia ratio (SHR), the vaccine gap caused by immune senescence, and the potential of metabolic modulators like GLP-1 receptor agonists to mitigate the pulmonary cytokine storm.

## 2. Materials and Methods

### 2.1. Search Strategy and Data Sources

**Figure 1:**
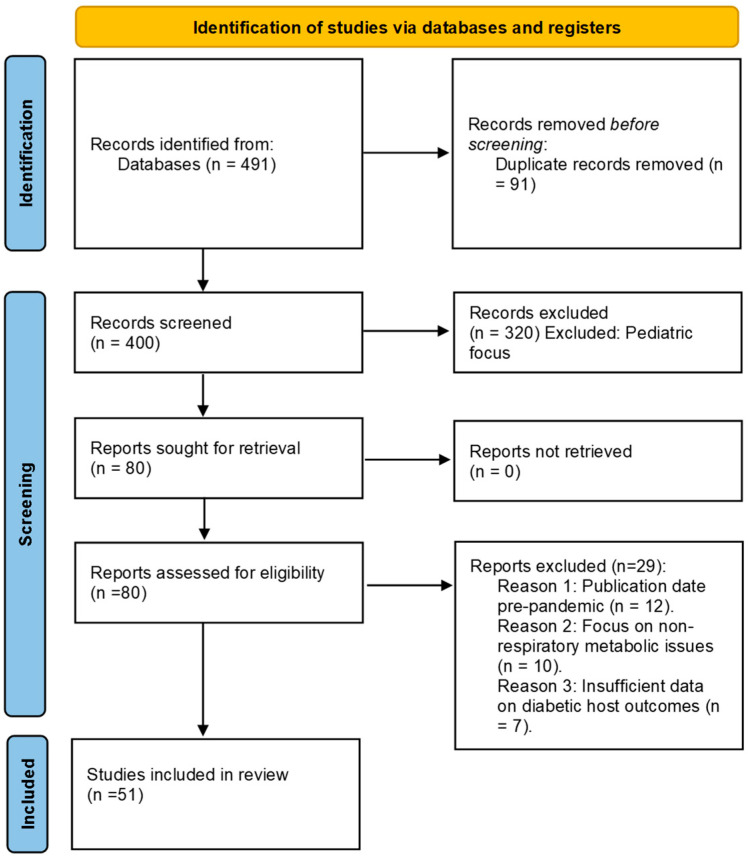
The diagram of the literature selection process.

To evaluate the evolving landscape of VRTIs in patients with DM during the post-pandemic era, a structured scoping review was performed across four major electronic databases: PubMed, Scopus, Embase, and Web of Science. The search was focused on articles published in the English language between January 2023 and February 2026 to capture the most recent clinical evidence and epidemiological shifts. The search strategy utilized a combination of Medical Subject Headings (MeSH) and the following search string: (“diabetes mellitus” OR “DM”) AND (“viral respiratory infections” OR “influenza” OR “SARS-CoV-2” OR “RSV” OR “hMPV” OR “Adenovirus”) AND (“pathophysiology” OR “outcomes” OR “management”). The search utilized Medical Subject Headings (MeSH) for PubMed, which were translated into Emtree terms for Embase. For Scopus and Web of Science, a combination of Boolean operators and free-text keywords was applied to titles, abstracts, and keywords. Data were extracted on 23 February, 2026. The initial search yielded 491 records. After removing 91 duplicates and screening 400 titles/abstracts, 80 full-text articles were assessed for eligibility, resulting in 51 studies included in this qualitative synthesis, as detailed in the PRISMA flow diagram (Figure 1). To ensure transparency, it is noted that a priori protocol registration was not performed for this review, which is acknowledged as a limitation. An extended search strategy is provided in the Supplementary Materials Table S1, File S1 and File S2.

### 2.2. Inclusion and Exclusion Criteria

Eligibility prioritized human studies focusing on adult or mixed adult-pediatric populations. We included the following:

- Observational cohorts, case-control studies, and randomized clinical trials.
- Clinical case series reporting novel metabolic complications (e.g., euDKA).
- Mechanistic investigations in humans that clarified the immunological interaction between hyperglycemia and viral virulence.

Studies restricted solely to animal models were excluded unless they provided essential mechanistic insights into human biological pathways that could not be otherwise observed.

### 2.3. Data Extraction and Synthesis

Following an initial screening of titles and abstracts for relevance, a full-text review of potentially eligible articles was conducted. For each included study, key data were extracted with a specific focus on

- Clinical risk and disease severity markers (e.g., hospitalization hazards and ICU admission rates).
- Immunological mechanisms (e.g., cytokine storm dynamics and surfactant protein dysfunction).
- Acute metabolic management strategies (e.g., the stress hyperglycemia ratio and SGLT2 inhibitor protocols).

### 2.4. Quality and Bias Mitigation

To mitigate potential selection bias inherent in the syntheses, we applied a defined search phrase and strict date range across multiple databases. While this review does not constitute a formal meta-analysis, the synthesis was structured to prioritize high-level evidence and recent registry data from the 2023–2026 respiratory seasons.

## 3. Results

### Epidemiology and Disease Burden

Recent data from the 2023–2026 respiratory seasons indicate that diabetic patients face a distinct vulnerability to pathogens previously considered primarily pediatric or opportunistic. The resurgence of hMPV and adenovirus in the adult diabetic population is partly attributed to the immunity debt following years of non-pharmaceutical interventions, summarized in Table 1. The lack of natural viral exposure between 2020 and 2023 resulted in a waning of mucosal IgA titers. In the diabetic host, who already suffers from accelerated immune senescence, this protection gap has lowered the threshold for these traditionally pediatric pathogens to invade the lower respiratory tract [8,9].

**Table 1:** Comparative Burden of Respiratory Viruses in Patients with Diabetes.

Viral Pathogen/Source	Specific Impact on Diabetes (DM)	Key Statistical Findings (Risk/Hazard)
Human Metapneumovirus (hMPV)/*The Journal of Infectious Diseases* (2025) [10]	Mortality equivalence to influenza: Recent matched cohort analysis (2025) confirms that hMPV in older adults (often with comorbidities like DM) carries the same hospitalization and mortality risk as influenza A/B and RSV	HR 1.00 (95% CI: 0.67–1.49) for hospitalization hMPV vs. influenza HR 0.79 (95% CI: 0.48–1.29) for mortality hMPV vs. influenza HR 1.00 (95% CI: 0.66–1.51) for hospitalization hMPV vs. RSV HR 0.93 (95% CI: 0.55–1.56) for mortality hMPV vs. RSV
Adenovirus/*NIH/PMC* (2025) [11]	Renal and respiratory failure: In adult inpatients, DM is a robust independent risk factor for severe outcomes, specifically AKI and respiratory failure, at rates significantly higher than seasonal influenza.	High Risk: Significant independent association between metabolic comorbidities and inpatient mortality/AKI compared to influenza cohorts (*p* < 0.001).
RSV/*The Journal of Infectious Diseases* [12]	Hospitalization Burden: DM is a top-tier driver of severe RSV requiring hospitalization in adults, often surpassing the risk seen in those with chronic liver disease or asthma	2- to 4-fold increased risk (Rate Ratio ~4.0) of hospitalization for adults with DM compared to controls.
SARS-CoV-2 vs. Influenza/*BMC Cardiovascular Diabetology* [13]	Mortality Gap: Direct head-to-head registry comparison showing that while DM worsens outcomes for both, COVID-19 remains significantly more lethal for diabetic patients than influenza	HR 2.81 (95% CI: 2.59–3.06) for death in type 2 diabetes patients with COVID-19 vs. influenza.
General Viral Impact/*BMJ Open* (2025) [14]	Systemic inflammation: Systematic review confirming that DM increases the risk of complications	OR 3.69 (95% CI: 2.75–4.94) for acute kidney injury in diabetic viral patients.

## 4. Pathophysiology and the Severity Gap

### 4.1. The Two-Hit Hypothesis

The disproportionate severity of VRTIs in the diabetic host is best characterized by a two-hit hypothesis that transforms a typical viral infection into a systemic crisis [15,16]. While the mechanisms described below, including surfactant protein glycosylation, adipose tissue sequestration, and gut–lung axis alterations, summarized in Table 2 are supported by emerging mechanistic literature and preclinical models, they remain largely theoretical and require further validation through definitive human mechanistic data.

- Hit 1: Chronic Immune Paralysis (The Primed State): Long before viral exposure, chronic hyperglycemia induces structural and functional impairments. This includes reduced pulmonary surfactant proteins A and D (SP-A and SP-D), which are critical for early viral neutralization. High glucose concentrations interfere with the carbohydrate recognition domain of SP-D, essentially leaving the alveolar epithelium exposed to direct viral entry. In addition, chronic metabolic stress induces metabolic memory via epigenetic reprogramming (e.g., histone methylation), keeping innate immune cells in a hyper-responsive, proinflammatory state.

A primary driver of the “Hit 1” primed state is the biochemical impairment of pulmonary surfactant proteins, specifically Surfactant Protein-D (SP-D). SP-D is a collectin essential for the innate immune defense of the lung, utilizing its carbohydrate recognition domain (CRD) to bind and neutralize viral pathogens. Under conditions of chronic hyperglycemia, high glucose concentrations lead to the non-enzymatic glycosylation of the CRD. This structural modification creates a physical and electrochemical barrier that prevents the SP-D from recognizing and binding to viral surface glycoproteins. In consequence, the virus bypasses this initial layer of mucosal immunity, facilitating direct entry into the alveolar epithelium and accelerating the progression from an upper respiratory infection to severe pneumonia [17,18,19,20].

- Hit 2: Acute Metabolic Decompensation (The Viral Trigger): Once the virus invades, the resulting cytokine storm, fueled by baseline meta-inflammation, triggers a surge in counter-regulatory hormones like cortisol and catecholamines. This precipitates acute insulin resistance and a glycemic gap that fuels further viral replication and endothelial dysfunction, leading to immunothrombosis and ARDS [21,22].

**Table 2:** The pathophysiological synergy.

Mechanism	Description of Dysfunction	Clinical Consequence	Supporting Evidence
Surfactant Glycosylation	Hyperglycemia disrupts SP-D carbohydrate recognition.	Facilitates direct viral entry into alveolar cells.	[16,23,24,25]
Metabolic Memory	Epigenetic histone methylation (H3K4me1).	Persistent pro-inflammatory state even after glucose normalization.	[18,19,20,26,27,28]
Immunothrombosis	Hyperglycemia-induced NETosis and platelet activity.	High risk of microvascular thrombosis and ARDS.	[15,17,22,25,29]

### 4.2. Metabolic Memory

The interaction between diabetes and viral pathogens represents a true syndemic, where the biological interface of metabolic dysfunction and viral virulence is mutually reinforcing. Clinical observations indicate that even after achieving euglycemia during hospitalization, diabetic patients continue to experience higher rates of ARDS and multiorgan failure. A critical finding in the literature is the role of metabolic memory, which explains why diabetic patients remain at higher risk even after achieving euglycemia during hospitalization. This phenomenon is driven by epigenetic imprinting: chronic hyperglycemia induces histone methylation (H3K4me1). This modification keeps innate immune cells in a hyper-responsive, pro-inflammatory state. In consequence, when a viral second hit occurs, the resulting cytokine storm is significantly more intense and less responsive to traditional glucose-lowering interventions, as the cellular memory of previous metabolic stress continues to drive inflammatory signaling [26,28].

### 4.3. Adipose Sequestration

The syndemic is complicated by diabesity. In the post-pandemic era, adipose tissue has been identified as a functional endocrine organ that sequesters viral particles, particularly SARS-CoV-2 and adenovirus. These cells express high levels of viral entry receptors, allowing the virus to persist within the fat depot, which leads to prolonged viral shedding and a continuous systemic inflammatory stimulus. Hypertrophic adipocytes in DM patients secrete elevated levels of leptin and reduced adiponectin, which directly signals the pulmonary endothelium to upregulate adhesion molecules, thereby predisposing the lung environment to the neutrophil-mediated immunothrombosis [27,28,29].

This dysmetabolic signaling directly targets the pulmonary endothelium, upregulating adhesion molecules and priming the lung for the neutrophil-led immunothrombosis that characterizes severe respiratory failure in this population. This sequestration mechanism offers a compelling biological explanation for the increased risk of Long COVID and delayed recovery observed in metabolically compromised hosts [30,31,32].

### 4.4. Gut–Lung Axis

The interaction between diabetes and viral respiratory infections is a multi-organ syndemic involving the gut–lung axis (GLA) and adipose tissue. This bidirectional pathway relies on microbial metabolites, specifically short-chain fatty acids (SCFAs) and desaminotyrosine, which act as systemic signaling molecules to prime pulmonary Type I interferon (IFN) responses. In a healthy state, the gut microbiome acts as a remote sentinel; however, the diabetic host exhibits reduced microbial diversity and significant dysbiosis. This leads to diminished production of Microbial-Associated Molecular Patterns (MAMPs), including Toll-like receptor (TLR) and NOD-like agonists, which directly reduces antiviral pulmonary immunity [33,34].

This dysbiosis creates a dangerous feedback loop where viral infection and antibiotic use [35] exacerbate intestinal permeability (“leaky gut”), facilitating bacterial translocation and increasing the risk of secondary bacterial pneumonia, a major cause of mortality [36,37].

## 5. Clinical Features and Prognostic Markers

The initial presentation is often similar to the general population (cough, fever, and dyspnea), metabolic comorbidities fuel a distinct phenotype prone to ARDS, multiorgan failure, and increased risk for pulmonary fibrosis. Clinicians should pay attention to gastrointestinal symptoms, specifically nausea and diarrhea, which precede, in some cases, lower respiratory tract signs and fever. Diagnosis might be complicated in the older adult, where the common classic viral presentation is often replaced by geriatric syndromes such as delirium, falls, or unexplained weakness [2].

Viral pneumonia remains the predominant severe manifestation [32]. RSV poses an equal threat to older adults with metabolic and cardiopulmonary comorbidities. When compared to influenza, hospitalized RSV patients often exhibit similar poor clinical outcomes, characterized by respiratory failure, increased dependence on mechanical ventilation and mortality [30]. In these patients, the progression to ARDS is a dominant threat.

### 5.1. The Stress Hyperglycemia Ratio (SHR)

Standard admission glucose is a limited prognostic tool because it fails to distinguish between acute stress and chronic poor glycemic control. Current evidence supports the use of the stress hyperglycemia ratio (SHR) [38,39,40,41], which provides a more accurate measure of the acute metabolic stress response by accounting for the patient’s baseline glycated hemoglobin (HbA1c). The SHR is calculated using the following formula:

$$SHR=\frac{Admission\;Glucose(mg/dL)}{Estimated\;Average\;Glucose(derived\;from\;HbA1c)}$$

High SHR is an independent risk factor for adverse outcomes [42], including mortality and the need for intensive care, with an Odds Ratio significantly higher than absolute hyperglycemia alone. Recent observational data from the 2023–2026 respiratory season suggest that an SHR threshold of ≥1.14 may serve as a potential prognostic indicator [43]. Patients exceeding this value demonstrate a 3.5-fold increase in the risk of progression to mechanical ventilation compared to those with stable glycemia, regardless of their baseline HbA1c in current cohorts. This indicates that the acute metabolic swing is a more potent driver of pulmonary immunothrombosis than chronic poor glycemic control [22,44]. While these findings are promising, the use of SHR as a definitive therapeutic decision-making tool requires confirmation in prospective clinical trials.

Incorporating SHR into admission protocols allows clinicians to identify metabolically volatile patients who require aggressive insulin titration and closer monitoring for secondary bacterial second-hit infections [45].

The two primary formulas are utilized depending on the units used for glucose:

1. For glucose in mg/dL:

$$SHR=\frac{Admission\;Glucose(mg/dL)}{28.7\times HbA1c\left(\%\right)-46.7}$$

2. For glucose in mmol/L:

$$SHR=\frac{Admission\;Glucose(mmol/L)}{1.59\times HbA1c\left(\%\right)-2.59}$$

### 5.2. The Vaccine Gap

While vaccination is the primary defense, a significant vaccine gap exists in the diabetic cohort [4,46]. Chronic hyperglycemia accelerates immune senescence, leading to the following:

- Reduced Seroconversion: lower titers of neutralizing antibodies following standard mRNA or protein-based vaccine series
- Impaired Persistence: a more rapid decline in protective immunity compared to non-diabetic controls necessitates personalized booster schedules
- Recommendation: future guidelines should evaluate high-dose vaccine formulations, similar to those used for the elderly, specifically for patients with severe insulin resistance to overcome this immunological lag.

A precision-based approach should consider the routine use of post-vaccination antibody titer monitoring (e.g., anti-spike IgG for SARS-CoV-2 or hemagglutination inhibition titers for influenza) in high-risk diabetic individuals. Such longitudinal serological surveillance would allow clinicians to determine the optimal timing for personalized booster schedules.

### 5.3. Iatrogenic Vigilance: The SGLT2i Paradox

The use of SGLT2 inhibitors represents a modern clinical paradox. They offer long-term cardio-renal protection but become a liability during acute viral stress. The combination of viral-induced anorexia and catabolic stress can trigger euDKA. The Diagnostic Trap appears because blood glucose levels often remain below 200 mg/dL, and euDKA is frequently missed, leading to critical delays in fluid and insulin resuscitation. This presentation is often masked by the absence of severe hyperglycemia, leading to delayed diagnosis and increased morbidity [47,48,49].

## 6. Preventive and Therapeutic Strategies

**Figure 2:**
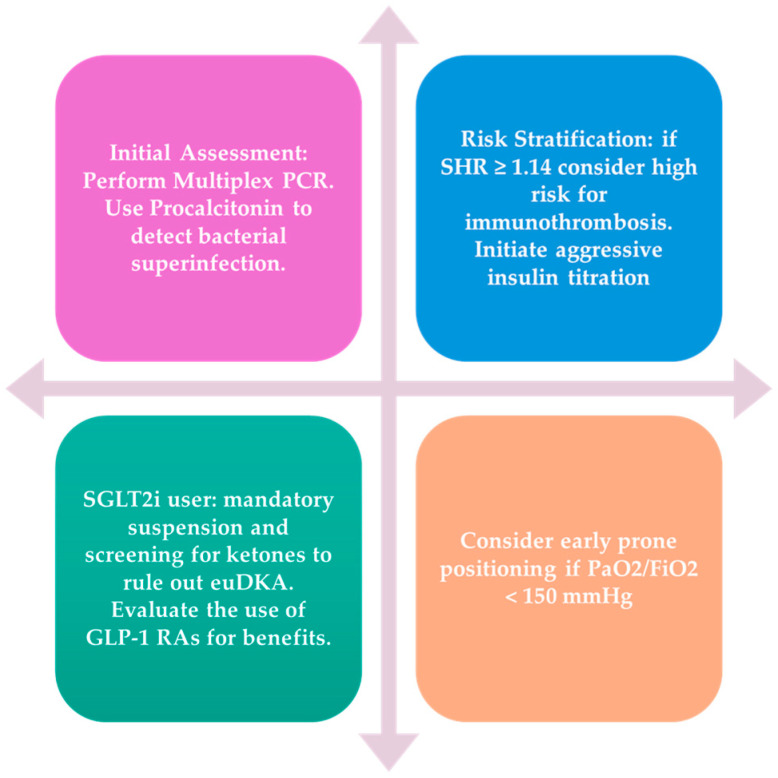
Precision Management Algorithm for the Diabetic Host with Acute Respiratory Viral Infection. This decision algorithm outlines the recommended triage and intervention workflow based on the stress hyperglycemia ratio (SHR) threshold of ≥1.14. It highlights the recommended suspension of SGLT2 inhibitors and the pharmacological pivot toward insulin and agents with anti-inflammatory potential (Metformin/GLP-1 RAs) in high-risk patients. PCR: Polymerase Chain Reaction; euDKA: Euglycemic Diabetic Ketoacidosis; GLP-1 RA: Glucagon-Like Peptide-1 Receptor Agonist.

To transition the findings of this review from theoretical risk factors into an actionable clinical framework, we propose a unified management algorithm (Figure 2). This pathway focuses on immediate risk stratification using the stress hyperglycemia ratio (SHR).

Upon initial presentation, a multiplex respiratory PCR panel (to identify the specific pathogen, e.g., influenza vs. hMPV) is combined with the calculation of the SHR. A critical threshold of SHR ≥ 1.14 might serve as the primary pivot point, categorizing patients into a high-risk phenotype prone to immunothrombosis and mechanical ventilation. The standard care for these high-risk patients must be superseded by aggressive metabolic correction, including the recommended suspension of SGLT2 inhibitors and immediate insulin titration. In accordance with the precision medicine approach advocated by our group, we recommend the use of procalcitonin-guided stewardship [50,51] to preempt the second hit of bacterial superinfection and, where clinically feasible, consider using the anti-inflammatory benefits of Metformin or GLP-1 RAs.

Management summarized in Table 3 requires a multi-layered defense: targeted immunoprophylaxis, metabolic modulation, and antiviral pharmacotherapy. Precision management requires a proactive shift in pharmacological priorities during the acute phase of infection.

The diabesity phenotype not only complicates the inflammatory response, but it also poses a significant pharmacological hurdle. The current literature highlights a knowledge gap regarding weight-adjusted dosing for antivirals such as Nirmatrelvir/Ritonavir or Oseltamivir. In patients with high BMI and type 2 DM, standard dosing may lead to sub-therapeutic plasma concentrations due to altered volume of distribution, potentially contributing to the severity gap observed in these cohorts. Until weight-stratified trials are available, clinicians should prioritize therapeutic drug monitoring or maintain high vigilance for treatment failure in patients with a BMI > 35.

Beyond systemic anti-inflammatory effects, GLP-1 receptor agonists (GLP-1RAs) have been shown to modulate the ACE/ACE2 balance in the pulmonary surfactant. By upregulating ACE2 expression, these agents may counteract the viral-induced downregulation of ACE2, thereby preventing the accumulation of Angiotensin II, a known driver of pulmonary vasoconstriction and fibroproliferative ARDS in diabetic hosts [21]. Preclinical models suggest that GLP-1 RAs (e.g., Liraglutide and Semaglutide) exert potent anti-inflammatory effects on the pulmonary endothelium, reducing cytokine release and enhancing surfactant production [52].

Early initiation of Metformin has demonstrated a remarkable ability to reduce the incidence of Long COVID [32]. This protection is likely mediated by the suppression of mTOR pathways and viral protein translation, alongside its anti-inflammatory properties via AMPK activation [25,52]. On the other side, clinicians must immediately suspend SGLT2 inhibitors upon hospitalization and resume dosage only after full recovery and stable oral intake.

**Table 3:** Precision Management Protocol for VRTIs in Diabetes (Framework).

Phase of Care	Intervention	Clinical Rationale and Target
Admission and Triage	Calculate Stress Hyperglycemia Ratio (SHR)	SHR may identify patients at high risk of immunothrombosis and mortality, independent of absolute glucose levels
[2,53]	Mandatory Respiratory Multiplex PCR Testing	Essential to identify viral coinfections, which correlate with higher ICU admission and mechanical ventilation rates
Pharmacological Adjustment [54]	Prudent Suspension of SGLT2 Inhibitors	Mitigates the potential risk of euglycemic diabetic ketoacidosis (euDKA) during states of catabolic stress and anorexia
[52]	Early Initiation of GLP-1 Receptor Agonists (investigational)	Hypothesized anti-inflammatory effects on pulmonary endothelium and enhancement of surfactant production
Acute Complication Management [55]	Procalcitonin-Guided Antibiotic Stewardship	Distinguishes viral-induced consolidation from bacterial second-hit superinfections (e.g., *S. aureus*) to prevent antibiotic overuse
[56]	Awake Prone Positioning (for PaO_2_/FiO_2_ < 150 mmHg)	Improves ventilation–perfusion (V/Q) matching in both COVID-19 and RSV-induced atelectasis
Metabolic Stabilization	Aggressive Insulin Titration During Corticosteroid Therapy	Corticosteroids used for ARDS exacerbate hyperglycemia; doses often require a >20–30% increase to maintain euglycemia
Post-Acute Follow-up	Longitudinal β-Cell Function Monitoring	Surveillance for new-onset diabetes or beta-cell exhaustion precipitated by viral pancreatic tropism and Long COVID

## 7. Discussion

The intersection of DM and VRTIs represents a syndemic whereby metabolic dysregulation and viral virulence amplify each other; also, a critical vaccine gap exists, since hyperglycemia reduces seroconversion rates and antibody persistence. Future research must determine if diabetic patients require high-dose vaccine formulations or more frequent boosters to overcome immune senescence.

Clinicians should consider implementing more prognostic precision tools like the stress hyperglycemia ratio (SHR), which has emerged as a superior prognostic marker over HbA1c or absolute admission glucose, acting as a functional tool to identify patients at risk of immunothrombosis and ARDS. Given the current lack of specific antiviral therapies for emerging threats such as hMPV and adenovirus, the SHR is not a simple biomarker tool; it becomes a primary clinical intervention target. In the absence of pathogen-directed drugs, managing the SHR acts as a functional antiviral strategy by modulating the metabolic host environment that otherwise fuels viral virulence and immunothrombosis.

The SHR threshold of ≥1.14 should be recognized as a definitive set value for patients at high-risk during admission triage. Patients crossing this critical tipping point face a 3.5-fold increased risk of progression to mechanical ventilation, regardless of their baseline HbA1c, necessitating immediate and aggressive insulin therapy to mitigate the acute metabolic decompensation.

In hospitalized patients, clinicians must maintain high suspicion for euDKA in patients treated at home with SGLT2 inhibitors, ensuring these agents are suspended during the acute phase of infection.

The potential anti-inflammatory roles of GLP-1 receptor agonists and the early use of Metformin to suppress viral protein translation offer promising avenues for reducing the burden of Long COVID and acute pulmonary damage. Although GLP-1RAs represent a promising therapeutic innovation for their ability to modulate the ACE/ACE2 balance and exert potent anti-inflammatory effects on the pulmonary endothelium, a significant evidence gap remains. Current support for their use in the acute phase of viral respiratory tract infections is derived primarily from preclinical models and retrospective observational data. Until large-scale, prospective randomized controlled trials specifically evaluate these agents in the context of acute viral stress, their use should be considered an emerging strategy rather than a standard of care for acute respiratory stabilization.

The vaccine gap in the diabetic cohort is fundamentally driven by hyperglycemia induced immune senescence, which manifests as impaired seroconversion and a rapid decline in neutralizing antibody titers. This immunological lag means that the diabetic population, even fully vaccinated with standard doses, may possess a lowered threshold for lower respiratory tract invasion. Future guidelines must investigate the utility of high-dose vaccine formulations and personalized booster schedules for those with severe insulin resistance.

Looking ahead, several critical evidence gaps must be addressed to refine the precision management of the diabetes–viral syndemic. While current antiviral dosing remains largely standardized, there is a clear clinical imperative for weight-stratified trials to determine if patients with a high BMI and type 2 diabetes require adjusted regimens to prevent sub-therapeutic plasma concentrations caused by an altered volume of distribution. Similarly, the promising anti-inflammatory and surfactant-modulating effects of GLP-1 receptor agonists observed in preclinical models now require rigorous validation through large-scale, prospective randomized controlled trials specifically designed for the acute phase of viral respiratory stress. Beyond acute care, future guidelines must investigate the utility of high-dose vaccine formulations and personalized booster schedules to overcome the immunological lag and rapid antibody decline characteristic of hyperglycemia-induced immune senescence. Finally, given the potential for viral pancreatic tropism and the acceleration of metabolic decline observed in post-acute phases, longitudinal surveillance of β-cell function is essential to manage the long-term risk of new-onset diabetes and chronic metabolic complications following severe respiratory illness.

## 8. Conclusions

The diabetic patient represents a unique immunological phenotype that cannot be managed with standard viral pneumonia protocols. The evidence synthesized in this review strongly suggests that the syndemic of diabetes and respiratory viruses is not merely additive, but mechanistic and synergistic. We are witnessing a distinct pathological entity where hyperglycemic immune paralysis, mediated by neutrophil dysfunction and surfactant glycosylation, transforms manageable viral heterogeneity into multiorgan failure. We need a fundamental restructuring of how we view preventative care, treating the metabolic susceptibility as aggressively as the virus itself.

The emerging superiority of the stress hyperglycemia ratio (SHR) over HbA1c underscores that acute glycemic control is not merely supportive care; it acts as a functional antiviral strategy, particularly for neglected pathogens like adenovirus or hMPV, for which no antiviral agents exist. Clinicians should focus on iatrogenic complications, specifically SGLT2 inhibitor-associated euglycemic ketoacidosis during the acute phase.

Vaccination constitutes the most effective mortality-reducing intervention; vaccines alone are insufficient in the setting of metabolic volatility. Future investigation must urgently address the vaccine gap on whether diabetic patients with severe insulin resistance require higher-dose vaccine formulations to overcome their immune senescence and mitigate vaccine hesitancy. Due to the long-term metabolic complications of viral infection, the acceleration of β-cell exhaustion seen in Long COVID needs longitudinal surveillance for the broader spectrum of respiratory pathogens. Closing the mortality gap requires precision medicine, treating the metabolic susceptibility with the same urgency as the viral pathogen itself.

## Data Availability

Not applicable.

## Supplementary Materials

The following supporting information can be downloaded at https://www.mdpi.com/article/10.3390/medicina62040770/s1. Table S1: Table of 51 included studies; Supplementary File S1: Detailed Search Strategy and Supplementary File S2: PRISMA-ScR.

## Abbreviations

ACE2	Angiotensin-Converting Enzyme 2
AKI	Acute Kidney Injury
AMPK	Adenosine Monophosphate-activated Protein Kinase
ARDS	Acute Respiratory Distress Syndrome
DM	Diabetes Mellitus
euDKA	Euglycemic Diabetic Ketoacidosis
GLA	Gut–Lung Axis
GLP-1RAs	Glucagon-Like Peptide-1 Receptor Agonists
HbA1c	Glycated Hemoglobin
hMPV	Human Metapneumovirus
IFN	Interferon
MAMPs	Microbial-Associated Molecular Patterns
mTOR	Mammalian Target of Rapamycin
NETosis	Neutrophil Extracellular Traps (process of formation)
PCR	Polymerase Chain Reaction
RSV	Respiratory Syncytial Virus
SCFA	Short-Chain Fatty Acids
SGLT2i	Sodium–Glucose Cotransporter-2 Inhibitors
SHR	Stress Hyperglycemia Ratio
SP-D	Surfactant Protein-D
VRTIs	Viral Respiratory Tract Infections
