# Supplementary material for: The Diabetes–Viral Respiratory Syndemic: Pathophysiological Insights and Precision Management: A Scoping Review
Source: Medicina (Kaunas). 2026 Apr 16;62(4):770. doi: 10.3390/medicina62040770 (PMC13117808; doi:10.3390/medicina62040770)
Supplement: Supplementary file 1 [file medicina-62-00770-s001.zip › S1 TABLE.pdf]

	Ref	Study (Author, Year)	Study Design	Key Population/ Sample Size	Key Finding for Diabetic Host
1.	[1]	Wang et al. (2025)	Pop-based Analysis	Adults >50 with DM (Europe) / n = 16,937	Lower vaccination coverage and higher hesitancy in diabetic cohorts
2.	[2]	Wildenbeest (2024)	Pop-based Analysis	Adults with RSV/SARS-CoV-2/Influenza / n = 32,960	Detailed poor outcomes in adults with metabolic comorbidities. the risk of acute cardiovascular complications in adults hospitalized for RSV vs COVID-19 or influenza.
3.	[4]	Bianchi et al. (2023)	Systematic Review/Meta	People with Diabetes / n = 4,076	Confirmed significantly higher vaccine hesitancy in the DM population
4.	[5]	Marchi et al. (2025)	Epidemiological Analysis	Post-COVID-19 Inpatients, SARI cases / n = 138	Shift in circulation of RSV and other pathogens in the post-pandemic era
5.	[6]	Surie et al. (2023)	Multicenter, IVY Network, 20 US States	Hospitalized Adults ≥60 / n = 5,784	Identified DM as a top-tier driver for severe RSV-associated hospitalization
6.	[7]	Siddiqi et al. (2025)	Pop-based Analysis	Heart Failure/Myocarditis patients related death / n = 33,750	DM isolated as an independent risk factor for ARDS and AKI during viral stress.
7.	[8]	Wang & Cao (2025)	Systematic Review/Meta	Patients with DM / n = 454,485	Linked glycemic variability to emerging viral complications in DM
8.	[9]	Sami et al. (2025)	Post-COVID Follow-up	Post-COVID-19 Patients / n = 96	Analyzed the persistence of glycemic dysregulation 6 months post-infection
9.	[10]	Bhaskar et al. (2025)	Matched Cohort	Nursing Home Residents / n = 919	NH residents are at high risk of severe outcomes and death within 90 days from hMPV, influenza, or RSV.
10.	[11]	Kwok et al. (2025)	Retrospective Cohort	Adult Inpatients / n = 41,206	DM is an independent risk factor for severe Adenovirus respiratory infection
11.	[12]	Osei-Yeboah (2024)	Modeling Study	Adults (2 European Countries) n = N/A	DM carries a 4-fold increased risk (Rate Ratio ~4.0) for RSV hospitalization
12.	[13]	Kristófi et al. (2022)	Nationwide Registry	T1DM and T2DM Patients / n = 16,116	COVID-19 significantly more lethal than Influenza in diabetic patients (HR 2.81)
13.	[14]	Zhang et al. (2025)	Systematic Review/Meta	Patients with Diabetes / n = 1,154,674	Complications in diabetic patients with viral infections; the increased rates of mortality and acute kidney injury
14.	[15]	Kombe Kombe et al. (2024)	Pathogenesis Review	Viral Respiratory Infections / n = N/A	Detailed the role of meta-inflammation in fueling the viral cytokine storm.
15.	[16]	Shireen Jawed et al. (2024)	Systematic Review	DM & Oxidative Stress / n = N/A	Hyperglycemia disrupts SP-D carbohydrate recognition, aiding viral entry.
16.	[17]	Arab et al. (2025)	Systematic Review	Long COVID Patients / n = N/A	Cardiopulmonary crosstalk evidence in post-acute metabolic recovery
17.	[18]	Dai et al. (2025)	Metabolic Modeling	Pulmonary Fibrosis / n = N/A	Targeting alveolar cell metabolism as a strategy for VRTI recovery
18.	[19]	Thieulent et al. (2025)	Preclinical Model	Diabetic Host Simulation / n = N/A	T2DM influences local pulmonary host responses and the CD4+ T lymphocyte-mediated immune response, leading to an increase in viral replication and a delay in virus clearance
19.	[20]	Zhong et al. (2025)	Immunological Review	Innate Antiviral Immunity / n = N/A	Metabolic regulation of interferon-mediated defenses in high-glucose states.

20.	[22]	Batista et al. (2025)	Narrative Review	Thromboinflammation / n = N/A	High risk of microvascular thrombosis and ARDS in diabetic VRTI
21.	[23]	Shamim et al. (2024)	Narrative Review	innate and adaptive immunity / n = N/A	Surfactant protein D: an immune surveillance
22.	[28]	Liu et al. (2025)	Review/Perspectives	Diabetes Complications / n = N/A	Trained immunity and epigenetic memory drive hyper-responsive inflammation
23.	[26]	Daskalaki et al. (2025)	Mechanistic Review	Macrophage Memory / n = N/A	Epigenetic histone methylation keeps cells in a pro-inflammatory state
24.	[27]	Perakakis et al. (2023)	Lanc. Diab. Review	Viral & Metabolic Disease / n = N/A	Bidirectional relationship where viruses trigger metabolic crises
25.	[30]	Peña-López et al. (2024)	Intensive Care Review	Severe RSV Disease / n = 12,447	DM increases dependence on mechanical ventilation in severe RSV
26.	[31]	Martin (2025)	Treatment Overview	Long COVID / n = N/A	Highlighted lack of "silver bullets" in post-viral metabolic recovery
27.	[32]	Khamidullina et al. (2024)	Retrospective Study	Hospitalized T2DM Patients / n = 2,486	Long-term glycemic decline observed after severe respiratory illness
28.	[33]	El-Khoury et al. (2025)	Narrative Review	VRTI & Microbiota / n = N/A	Reduced microbial diversity in DM diminishes antiviral pulmonary immunity
29.	[34]	Eladham et al. (2024)	Mechanistic Review	Gut-Lung Axis / n = N/A	Interaction between DM dysbiosis and severe respiratory outcomes
30.	[36]	Yu et al. (2025)	Review	Type 2 Diabetes / n = N/A	Pathogenesis of gut dysbiosis and its impact on systemic immunity
31.	[37]	Ou et al. (2023)	Mechanistic Review	Influenza A Cohorts / n = N/A	Gut microbiota's role in maintaining pulmonary immune balance
32.	[38]	Wang et al. (2026)	Sys Review/Meta	SHR / n = N/A	SHR outperforms absolute glucose in predicting severity/mortality
33.	[39]	Xie et al. (2026)	Sys Review/Meta	ICU Patients with Sepsis SHR / n = 37,790	SHR significantly associated with all-cause mortality in critical illness
34.	[40]	Zheng et al. (2026)	Machine Learning Cohort	Sepsis Patients, SHR / n = 1,834	Persistent stress hyperglycemia trajectories predict mortality
35.	[41]	Zhang et al. (2023)	MIMIC-IV Database	Critically Ill Patients, SHR / n = 3,887	Validated SHR as a superior prognostic marker in intensive care cohorts
36.	[42]	Ma et al. (2024)	Two Center Retrospective	Sepsis Patients, SHR / n = 1,835	Higher SHR linked to 3.5-fold increased risk for mechanical ventilation
37.	[43]	Aon et al. (2022)	Retrospective Study	COVID-19 Inpatients with DM / n = 395	SHR threshold of ≥1.14 serves as a critical clinical tipping point
38.	[44]	Rui et al. (2025)	Retrospective Study	Critically Ill T2DM SHR / n = 993	Confirmed the association between SHR and mortality in diabetes
39.	[45]	Hughes et al. (2025)	Preclinical Model	Cystic Fibrosis/DM Model / n = N/A	High glucose increases bacterial resistance during viral secondary hit
40.	[46]	Vega-Piris (2024)	Hospitalized Adults	Adults ≥65 years / n = 6,546	RSV severity in older adults with DM mimics COVID-19 outcomes
41.	[47]	Khedr et al. (2023)	Systematic Case Review	COVID-19 Patients / n = 12	SGLT2i use in acute viral stress linked to euglycemic DKA (euDKA)
42.	[48]	Kleinjan et al. (2024)	Case Report	Metabolic Patients / n = 2	Prudent suspension of SGLT2i during catabolic states is necessary
43.	[49]	Baek et al. (2024)	Real World Study	T2DM Patients / n = 110	Real-world evidence of euDKA triggered by SGLT2i during illness

44.	[50]	Liu et al. (2025)	Clinical Application	Sepsis/Infection Cohorts / n = 252	Procalcitonin-guided stewardship is vital to avoid secondary hits
45.	[51]	Sivgin et al. (2023)	Independent Risk Study	RSV and DM Patients / n = 46	DM and bacterial co-infection are primary drivers of RSV severity
46.	[52]	Ashique et al. (2025)	Critical Review	Patients with Diabetes / n = N/A	Evaluated Long COVID impacts and potential for Metformin protection
47.	[25]	Bie et al. (2025)	Microbiological Review	Viral Metabolism / n = N/A	Viruses "hijack" glucose metabolism, worsening diabetic outcomes
48.	[53]	Pak J. (2025)	Multiplex PCR Study	Diabetic/Non-Diabetic / n = N/A	Multiplex PCR essential for distinguishing viral from bacterial hits in DM
49.	[54]	Cardona et al. (2025)	Protocol Development	euDKA Protocols / n = N/A	Necessary management protocols for euglycemic DKA during illness
50.	[24]	Pedreañez et al. (2024)	Review Article	SP-D implication in viral infection / n = N/A	Role of the receptor for advanced glycation end products in the severity of SARS-CoV-2 infection in diabetic patients
51.	[29]	Zhou et al. (2026)	Review Article	Host response / n = N/A	Immunothrombotic storm in viral sepsis
