# Supplementary material for: The Diabetes–Viral Respiratory Syndemic: Pathophysiological Insights and Precision Management: A Scoping Review
Source: Medicina (Kaunas). 2026 Apr 16;62(4):770. doi: 10.3390/medicina62040770 (PMC13117808; doi:10.3390/medicina62040770)
Supplement: Supplementary file 1 [file medicina-62-00770-s001.zip › S2 Search.pdf]

Supplementary File S1: Detailed Search Strategy

The Diabetes-Viral Respiratory Syndemic: Pathophysiological Insights and Precision Management

Authors: Mihai A.M., Marc M., Lucaciu F., Sima A.

Review Type: Scoping Review

Search Period: January 1, 2023 – Early 2026

1. PubMed (NLM)

Search Date: February 23, 2026 **Strategy:** Utilized Medical Subject Headings (MeSH) and Title/Abstract [tiab] terms.

Search String: > ("Diabetes Mellitus"[MeSH Terms] OR "diabetes mellitus"[tiab]) AND ("Respiratory Tract Infections"[MeSH Terms] OR "Influenza, Human"[MeSH Terms] OR "SARS-CoV-2"[MeSH Terms] OR "Respiratory Syncytial Viruses"[MeSH Terms] OR "Adenoviridae"[MeSH Terms] OR "Metapneumovirus"[MeSH Terms] OR "viral respiratory infection"[tiab] OR "RSV"[tiab] OR "hMPV"[tiab] OR "adenovirus"[tiab]) AND ("pathophysiology"[tiab] OR "treatment outcome"[MeSH Terms] OR "outcomes"[tiab] OR "disease management"[MeSH Terms] OR "management"[tiab])

Filters: English language, Publication date from 2023/01/01 to 2026/02/23.

2. Embase (Elsevier)

Search Date: February 23, 2026 **Strategy:** Adapted MeSH terms to **Emtree** keywords and used field tags for title/abstract (:ti,ab).

Search String: > ('diabetes mellitus'/exp OR 'diabetes mellitus':ti,ab) AND ('respiratory tract infection'/exp OR 'influenza'/exp OR 'sars-cov-2'/exp OR 'respiratory syncytial virus'/exp OR 'adenovirus'/exp OR 'human metapneumovirus'/exp OR 'viral respiratory infection':ti,ab) AND ('pathophysiology':ti,ab OR 'clinical outcome'/exp OR 'outcomes':ti,ab OR 'disease management'/exp OR 'management':ti,ab)

Filters: English language, 2023–2026.

3. Scopus and Web of Science

Search Date: February 23, 2026 **Strategy:** Employed Boolean operators (AND/OR) with broad keyword coverage in Title, Abstract, and Keywords (TITLE-ABS-KEY or TS).

Search String: > (TITLE-ABS-KEY("diabetes mellitus") AND TITLE-ABS-KEY("viral respiratory infection" OR "influenza" OR "SARS-CoV-2" OR "respiratory syncytial virus" OR "RSV" OR "human

metapneumovirus" OR "hMPV" OR "adenovirus") AND TITLE-ABS-KEY("pathophysiology" OR "outcomes" OR "management"))

Filters: Language: English; Date: 2023–2026.

Parameter,Selection Criteria

Databases,"PubMed, Scopus, Embase, Web of Science "

Language,English

Timeframe,January 2023 – Early 2026

Primary Keywords,"Diabetes mellitus, viral respiratory infections, influenza, SARS-CoV-2, RSV, hMPV, Adenovirus, pathophysiology, outcomes, management "
